# Micellar liquid chromatographic method for the simultaneous determination of Levofloxacin and Ambroxol in combined tablets: Application to biological fluids

**DOI:** 10.1186/1752-153X-7-162

**Published:** 2013-10-01

**Authors:** Fathalla F Belal, Mohie K Sharaf El-Din, Nahed M El-Enany, Samar Saad

**Affiliations:** 1 Department of Analytical Chemistry, Faculty of Pharmacy, University of Mansoura, Mansoura 35516, Egypt

**Keywords:** HPLC, Micellar, Simultaneous determination, Levofloxacin (LEV) Ambroxol (AMB), Co-formulated tablets, Human plasma

## Abstract

**Background:**

Levofloxacin hemihydrate (LEV) and ambroxol HCl (AMB) are available for the treatment of upper and lower respiratory tract infections. A survey of the literature reveals that two reversed phase HPLC methods were e reported for the simultaneous determination of LEV and AMB in pharmaceutical preparations. However the reported methods suffers from the low sensitivity, no application of the method in the combined tablets and no application to biological fluids. Also the toxic effects of the used solvents which are harmful to human beings. For this reason, our target was to develop a simple sensitive, less hazardous micellar HPLC method for the simultaneous determination of LEV and AMB in their combined dosage forms and plasma.

**Results:**

The method showed good linearity over the ranges of 1–44 μg/mL and 1–20 μg/mL with limits of detection 0.26 and 0.07 μg/mL and limits of quantification 0.80 and 0.20 μg/mL for LEV and AMB, respectively. The method was further extended to the determination of LEV in spiked human plasma with mean percentage recoveries of 100.10% ± 1.14 as well as determination of LEV in real human plasma without prior extraction. Statistical evaluation of the data was performed according to ICH Guidelines.

**Conclusion:**

The suggested method was successfully applied for the simultaneous analysis of the studied drugs in their co-formulated tablets and human plasma. The mean percentage recoveries in combined tablets were 100.20 ± 1.64 and 100.72 ± 1.11 for LEV and AMB, respectively and 100.10 ± 1.14 for LEV in spiked human plasma. Statistical comparison of the results with those of the comparison method revealed good agreement and proved that there were no significant difference in the accuracy and precision between the two methods respectively.

## Background

MLC is a mode of reversed phase liquid chromatography (RPLC), in which the mobile phases are aqueous solutions of a surfactant at a concentration above the critical micellar concentration (cmc). Anionic sodium dodecyl sulphate (SDS) is the most widely used surfactant in MLC, but neutral Brij-35 or cationic N-cetyltrimethylammonium chloride are also used. In these media, the great variety of interactions between the solutes, micelles and stationary phase makes MLC a highly versatile technique, which is appropriate for a wide range of solutes (hydrophilic and hydrophobic compounds) that can be separated in the same run.

Most procedures for the determination of compounds by MLC make use of micellar mobile phases containing an organic modifier (hybrid micellar mobile phases), which is usually a short-chain alcohol (methanol, propanol, butanol or pentanol) or acetonitrile. These modifiers increase the elution strength and often improve the shape of the chromatographic peaks. The modifiers solvate the bonded stationary phase and reduce the amount of surfactant adsorbed, the effect becoming larger as the concentration and the hydrophobicity of the alcohol increases. Selection of the pH of the mobile phase is also often important for the resolution of complex mixture, because of the side acid–base reactions of many solutes [1].

**Figure 1:**
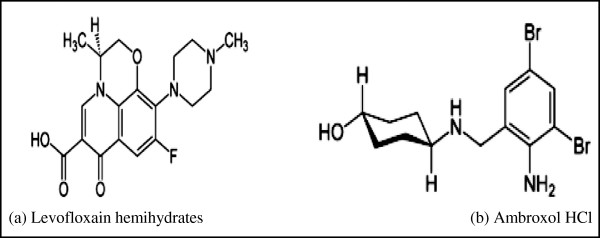
**The structural formulae of the studied drugs. (a)** Levofloxain hemihydrates and **(b)** Ambroxol HCl.

Levofloxacin hemihydrate (LEV) Figure 1a, (−)-S-9-fluoro-2,3-dihydro-3-methyl-10-(4-methyl)-1-piperazinyl)-7-oxo-7H-pyrido[1,23-de]-1,4-benzooxazine-6-carboxylic acid is second generation fluoroquinolones [2]. It is the S-(−)-isomer of ofloxacin [2] and acts as antibacterial by inhibiting DNA gyrase and topoisomerase IV enzyme [3]. It is the subject of a monograph in the United States pharmacopoeia USP [3].

Ambroxol hydrochloride (AMB) Figure 1b, trans-4-(2-amino-3, 5-dibromobenzylamino)cyclohexanol hydrochloride [2] is used as mucolytic expectorant [4]. The drug is the subject of a monograph in the British pharmacopoeia BP [5].

The literature revealed many methods for the determination of LEV; including a review on the spectrophotometric methods for its determination up to 2008 [6], other specrophotometric methods [7-9], spectrofluorometric methods [10,11], HPLC with UV detection [12,13], fluorometric detection [14], tandem mass spectrometric detection [15]. Capillary electrophoresis with electrochemiluminescence detection [16], electrochemical methods [17], chemiluminescence [18] and flow injection analysis with UV, potentiometric and conductometric detection [19] were also reported.

Regarding AMB: several methods were also described for its determination either per se or in pharmaceutical preparations;including potentiometric titration [5] using alcohol as a solvent, adding 0.01 M hydrochloric acid and titration using 0.1 M sodium hydroxide. AMB was determined using spectophotometric methods [20,21], stability indicating HPTLC [22]. HPLC with UV detection [23-25], mass spectrometric detection [26], potentiometric detection [27], amperometric detection [28], GC [29], flow injection analysis [30,31], micellar electrokinetic capillary chromatographic method [32] and electrochemical methods [33,34].

Both drug were simultaneously determined by HPLC [35], TLC [36] and UV spectrophotometry [37]. A fixed dose of LEV and AMB is available for the treatment of upper and lower respiratory tract infections.

To the best of our knowledge no method has been reported concerning the analysis of such mixture using a micellar liquid chromatographic method, a mobile phase containing surface active agent (SAA) was useful for the analysis of LEV in spiked and real human plasma, since SAA dissolves the amino acids present in human plasma, therefore no need for prior extraction step which is time consuming and decrease the hazardous effect of using organic solvent.

In the present work, a micellar HPLC method with UV detection was utilized for the simultaneous analysis of LEV and AMB with good resolution within retention times less than 6 min. This method could be applied for the quantitative determination of the studied drugs in their and prepared co-formulated tablets, as well as determination of LEV in spiked human plasma. The results obtained were promising.

## Materials and methods

### Apparatus

•Chromatographic separation was carried out using a Merck Hitachi Chromatograph model L-7100 equipped with a Rheodyne injector valve with a 20 μL loop, and a Merck Hitachi L-7400 UV detector operated at 220 nm. The chromatograms were recorded on a Merck Hitachi D-7500 integrator. Mobile phase was filtered using Millipore filter Sibata and degassed using Merck solvent L-7612 degasser.

•A Consort P-901 pH-meter was used for pH measurements.

•Ultrasonic bath, model SS 101 H 230, USA.

### Materials and reagents

All the chemicals used were of Analytical Reagent Grade, and the solvents were of HPLC grade.

•LEV was kindly provided by EUROPEAN EGYPTIAN PHARMACEUTICALS company, batch # KYLFAM20090605B.

•AMB was kindly provided by GlaxoSmithkline, S. A. E. Elsalam city, Egypt, batch # VBNOB2011.

•Furosemide (FUR), used as the internal standard (IS), was kindly donated by Alexandria CO. for Pharmaceuticals, Alexandria, Egypt.

•Leeflox® tablets, manufactured by Pharonia Pharmaceuticals New Borg EL-Arab city, Egypt, batch # 1131002, labeled to contain 250 mg LEV.

•Ambroxol® tablets, Manufactured by GlaxoSmithkline, S. A. E. Elsalam city, Egypt, batch # 1020144, labeled to contain 30 mg AMB.

•Sodium dodecyl sulphate (SDS) 90%, triethylamine (TEA) and orthophosphoric acid 85% were obtained from Riedel-deHäen (Sleeze, Germany).

•Methanol, n-propanol and acetonitrile (HPLC grade) were obtained from Sigma- Aldrich (Germany).

•Human plasma was kindly provided by Mansoura University Hospitals, Mansoura, Egypt and kept frozen (−5°C) until used after gentle thawing.

### Chromatographic conditions

Column: Spherisorb-ODS 2 C18 column (150 mm × 4.6 mm i.d., 5 μm particle size) Shimadzu, Kyoto, Japan. The column hold up value was the first deviation of the base line obtained.

Mobile phase: a solution consists of 0.15 M SDS, 8% n-propanol, 0.3% TEA, prepared in 0.02 M orthophosphoric acid. The pH of the mobile phase was adjusted to pH 4.0 using orthophosphoric acid and the flow rate was 1 mL/min.

The column was operated at room temperature and the wavelength was monitored at 220 nm. FUR was selected as the internal standard since it gave good resolution with both LEV and AMB.

### Standard solutions

Stock solutions of 200 μg/mL of LEV, 200 μg/mL of AMB and 200 μg/mL of FUR (IS) were prepared by dissolving 20.0 mg of LEV, AMB and of FUR separately in 100 mL of methanol with the aid of an ultrasonic bath. Working standard solutions were prepared by appropriate dilution of the stock solutions with methanol. Standard laboratory prepared mixture solutions were prepared by mixing appropriate volumes of LEV and AMB stock solutions in 50 mL volumetric flasks and diluting to the volume with methanol keeping the medicinally recommended ratios of 25: 6 for LEV and AMB, respectively. All solutions were stored in the refrigerator and found to be stable for at least 10 days without alteration.

### Procedures

#### Construction of calibration graphs

Accurately measured aliquot volumes of the suitable drug working standard solutions were transferred into a series of 10 mL volumetric flasks so that the final concentration was in the range of 1–44 μg/mL for LEV and 1–20 μg/mL for AMB. To each flask, 8 μg/mL (final concentration) of FUR standard solution was added as internal standard. Then, the solutions were completed to the volume with the mobile phase. Aliquots of 20 μL were injected (triplicate) and eluted with the mobile phase under the optimum chromatographic conditions. The average peak area ratio (Drug/I.S.) versus the final concentration of the drugs in μg/mL was plotted. Alternatively, the corresponding regression equations were derived.

### Analysis of LEV/AMB Laboratory prepared mixtures

Aliquots of LEV and AMB standard laboratory prepared mixture solutions were transferred into a series of 10-mL volumetric flasks. To each flask, 8 μg/mL (final concentration) of FUR standard solution was added as internal standard. Then, the solutions were completed to the volume with the mobile phase. The solutions were diluted to the mark with the mobile phase and mixed well. The above procedure described under “Construction of the Calibration Graphs” was then performed. The percentage recoveries were calculated by referring to the calibration graphs, or using the corresponding regression equations.

### Analysis of LEV and AMB in their single tablets

An accurately weighed quantity of the mixed contents of 10 powdered Leeflox® or Ambroxol® tablets equivalent to 20.0 mg of LEV and AMB, respectively were transferred separately into a 100 mL volumetric flasks and 80 mL of methanol were added. The contents of the flask were sonicated for 30 min, completed to the volume with the same solvent, mixed well and filtered. Aliquots containing suitable concentrations of the studied drugs were analyzed as described under “construction of calibration graphs”. The nominal content was calculated either from a previously plotted calibration graph or using the corresponding regression equation.

### Analysis of the studied drugs in their co-formulated tablets

Laboratory prepared tablets containing 250 mg of LEV and 60 mg of AMB were mixed with tablet excipients; lactose (15) mg, starch (15) mg, talc (20) mg and magnesium stearate (10) mg per each tablet. An accurately weighed quantity of the mixed contents of 10 prepared tablets equivalent to 25.0 mg LEV and 6.0 mg AMB (according to their pharmaceutical ratio) was transferred into 100 mL volumetric flasks and 80 mL of methanol were added. The contents of the flask were sonicated for 30 min, completed to the volume with the same solvent, mixed well and filtered. Aliquots containing suitable concentrations of the studied drugs over the working concentration range were analyzed as described under “construction of calibration graphs”. The nominal content was calculated either from a previously plotted calibration graphs or using the corresponding regression equations.

### Analysis of LEV in spiked human plasma

Aliquots of LEV working standard solution was transferred into a series of 10 mL volumetric flasks, so that its final concentration is in the range of 1–3 μg/mL. The contents of the flasks were diluted to about 8 mL with the mobile phase, to prevent plasma protein precipitation with methanol (solvent of LEV), 1 mL of human plasma was added to each flask, and the volumes were completed to the mark with the mobile phase and mixed well. Aliquots of 20 μL were injected (triplicate) and eluted with the mobile phase under the reported chromatographic conditions. A blank experiment was carried out simultaneously. The peak area was plotted versus the concentration of the drug in μg/mL.

### Procedure for patient samples

A healthy volunteer (female, 30 years old) had been administered Leeflox 750 mg® tablet after 10 hours of fasting. A blood sample was taken from the volunteer before administration of the tablets as a blank. Then, blood samples were collected at several time intervals after oral administration. The samples were drawn into test tubes containing EDTA as anticoagulant and centrifuged at 4000 rpm for 30 min. The supernatant plasma was transferred into test tubes. 1 mL aliquots of the supernatant plasma were transferred into a series of 10 mL volumetric flasks. The procedure described under the analysis of spiked human plasma was then followed.

## Results and discussion

**Figure 2:**
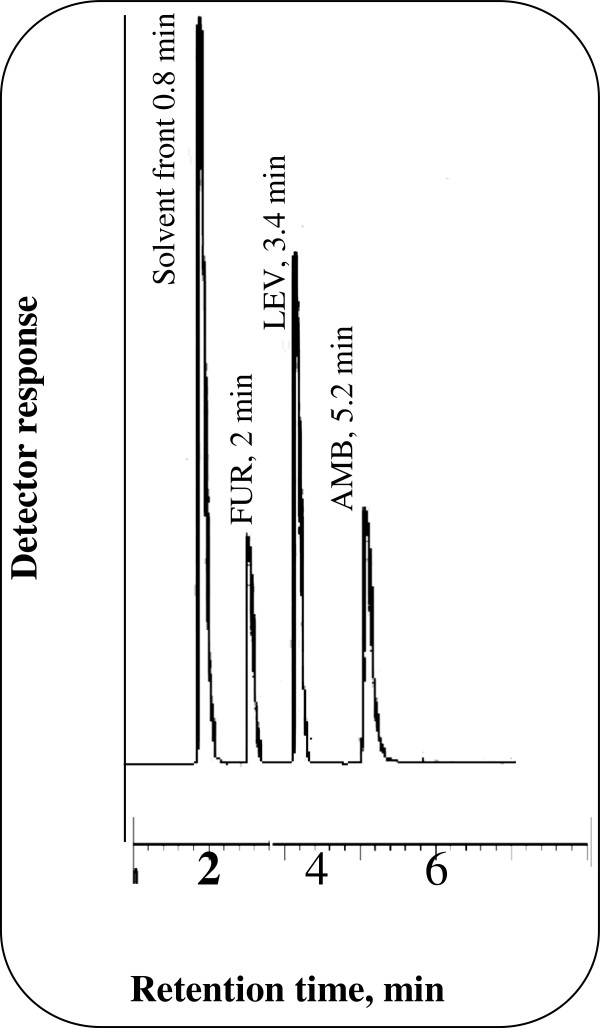
Typical chromatogram of synthetic mixture of 25 μg/mL LEV and 6 μg/mL AMB using 8 μg/mL FUR (I.S) in 0.15 M SDS, 0.3% triethylamine, 8% n-propanol all prepared in 0.02 M orthophosphoric acid at pH 4.0.

The proposed method permitted good separation of LEV and AMB with resolution factor (Rs) = 3.81 and selectivity factor (α) = 2.45 in a reasonable time less than 6 min. Figure 2 Illustrates a typical chromatogram for a laboratory prepared mixture of the two studied drugs under the described chromatographic conditions. The retention times for LEV and AMB were 3.4 and 5.2 min., respectively. The proposed method offers high sensitivity since 1 μg/mL of LEV and 1 μg/mL of AMB could be determined accurately. It also permitted the accurate analysis of the studied drugs in their co-formulated tablets and the analysis of LEV in spiked as well as in real human plasma.

### Optimization of the chromatographic performance and system suitability

Well-defined symmetrical peaks were obtained after thorough experimental trials that can be summarized as follows:

### Choice of column

Two different columns were used for performance investigations, including: Spherisorb-ODS 2 C18 column (150 mm × 4.6 mm i.d., 5 μm particle size) and Symmetry® C18 column (250 mm × 4.6 mm i.d., 5 μm particle size), The experimental studies revealed that the first column was the most suitable since it produced symmetrical peaks with high resolution. The second column was not suitable for the analysis since it resulted in delay in retention times, 10.3 min for LEV and 48 min for AMB.

### Choice of appropriate wavelength

Five wavelengths (220, 230, 248, 300 and 310 nm) were tried to detect the most suitable one for analysis and separation of both drugs. The UV detector response of both drugs was studied and the most suitable wavelength was found to be 220 nm showing the highest sensitivity with a reasonable response and good separation for both drugs.

### Mobile phase composition

Several modifications in the mobile phase composition were performed in order to study the possibilities of improving the performance of the chromatographic system. These modifications included the change of the type and % concentration of the organic modifier, the concentration of SDS and the pH. The results obtained are abridged in Table 1.

**Table 1:** Effect of experimental parameters on the number of theoretical plates, resolution and selectivity factor

**Parameter**		**Number of theoretical plates/m (N)**		**Resolution (R)**	**Selectivity factor(α)**
		**LEV**	**AMB**		
**% concentration of organic modifier (v/v)**	6	3390	5000	2.27	1.85
	8	3800	6880	3.81	2.05
	10	5620	3200	2.76	2.45
	12	3590	5350	2.98	2.42
	14	3350	6000	3.10	2.67
**Concentration of SDS, M**	0.05	4550	8250	5.54	2.68
	0.10	4390	6500	3.81	2.45
	0.12	4160	5730	3.14	2.20
	0.15	5610	3190	3.81	2.45
	0.16	3910	4960	2.44	2.09
	0.18	2750	3310	1.90	2.11
	0.20	2910	4570	1.75	1.84
**pH of the medium**	3	4630	7130	3.90	2.35
	4	4390	6500	3.81	2.45
	5	3370	6550	3.80	2.46
	5.5	3980	7340	3.57	2.35
	6	3160	6730	3.58	2.31
	7	1360	8440	2.99	2.31
**Flow rate (mL/min)**	0.6	3530	5210	2.09	1.90
	0.8	2960	4900	2.06	1.83
	1.0	4390	6500	3.81	2.45
	1.2	2790	3660	1.92	1.84
	1.4	2100	3340	1.89	1.85
	1.6	2230	3380	1.85	1.85
	1.8	1370	3550	2.10	1.88

### Type of organic modifier

Different organic modifiers were tried during the experimental study to choose the most suitable one for chromatographic separation of the two drugs. The studied organic modifiers included methanol, acetonitrile, n-propanol, 2-propanol and absolute ethanol. It was found that; using methanol and absolute ethanol showed overlapping of the studied drugs, while 2-propanol and acetonitrile showed slight overlapping, a little delay in retention times and decrease in number of theoretical plates, especially for AMB. In addition, methanol, ethanol, acetonitrile and 2-propanol showed lower sensitivity. So, n-propanol was the organic modifier of choice giving good resolved and highly sensitive peaks within a reasonable time (less than 6 min.).

### Concentration of organic modifier (%)

The effect of changing the % concentration of n-propanol on the selectivity and retention times of the test solutes was investigated using mobile phases containing concentrations of 6–14% of n-propanol. It was found that the retention times of both LEV and AMB decreased upon increasing the % concentration of n-propanol. The study revealed that the optimum chromatographic performance was achieved upon using 8% n-propanol regarding the resolution of the two drugs and number of theoretical plates. Concentrations less than 6% resulted in a broad and less sensitive peaks and it was time consuming, whereas concentrations higher than 14% decreased number of theoretical plates for both drugs.

### Concentration of SDS, M

The effect of changing the concentration of SDS on the selectivity and retention times of the test solutes was investigated using mobile phases containing a concentration of 0.05–0.2 M SDS. It was found that the retention times of both LEV and AMB decreased upon increasing the % concentration of SDS. The study revealed that the optimum chromatographic performance was achieved upon using 0.15 M SDS regarding the resolution of the two drugs and number of theoretical plates. Concentrations less than 0.05 M SDS resulted in great increase in the retention time, whereas concentrations higher than 0.2 M SDS decreased number of theoretical plates.

### pH

The effect of changing the pH of the mobile phase on the selectivity and retention times of the test solutes was investigated using mobile phases of pH ranging from 3–7. It was found that the retention times of both LEV and AMB didn’t greatly affected by the change of pH. However increasing pH greater than 4 resulted in decrease of number of theoretical plates of LEV. Table 1 illustrates that pH 4.0 was the most appropriate one yielding well resolved peaks and the highest number of theoretical plates.

### Flow rate

The effect of flow rate on the formation and separation of peaks of the studied compounds was investigated over the range of 0.6-1.4 mL/min. A flow rate of 1 mL/min. was optimal for highest plate count and good separation in a reasonable time, Table 1.

### The nature of internal standard

Different internal standards such as triclabendazole, spironolactone, xipamide, trimethoprim and furosemide were investigated. Furosemide was the internal standard of choice as it produced highest resolution factor and good separation from the peaks of the two drugs.

### Method validation

#### Linearity and range

Under the above described experimental conditions, a linear relationship was established by plotting the peak area ratio [drug/I.S.] against the drug concentration in μg/mL. The concentration range was found to be 1–44 μg/mL for LEV and 1–20 μg/mL for AMB. Linear regression analysis of the data gave the following equations:

$$\mathrm{PA}=\;‒\;0.0185+0.0682C\left(r=0.9999\right)\;\mathrm{for}\;\mathrm{LEV}$$

$$\mathrm{PA}=\;‒\;0.0370+0.0748C\left(r=0.9999\right)\;\mathrm{for}\;\mathrm{AMB}$$

Where: P is the peak area ratio, C is the concentration of the drug in μg/mL and r is the correlation coefficient.

The high values of the correlation coefficients with small intercept indicate the good linearity of the calibration graph.

Statistical analysis [38] of the data gave high value of the correlation coefficient (r) of the regression equations. Small values of the standard deviation of residuals (S_y/x_), of intercept (S_a_), and of slope (S_b_) indicate low scattering of the points around the calibration curves. Also, small values of the percentage relative standard deviation (RSD %) and the percentage relative errors (% Er) indicate high accuracy and high precision of the proposed method, Table 2.

**Table 2:** Analytical performance data for the determination of the LEV and AMB by the proposed method

**Parameter**	**LEV**	**AMB**
Linearity range (μg/mL)	1- 44	1-20
Intercept (*a*)	−0.086	−0.037
Slope (*b*)	0.0682	0.0748
Correlation coefficient (*r*)	0.9999	0.9999
SD of residuals (S_*y/x*_)	8.5 × 10^-3^	1.9 × 10^-3^
SD of intercept (S_*a*_)	5.5 × 10^-3^	1.5 × 10^-3^
SD of slope (S_*b*_)	2.00 × 10^-4^	1.00 × 10^-4^
Percentage relative standard deviation, % RSD	0.762	1.425
Percentage relative error, *%* Error	0.288	0.640
Limit of detection, LOD (μg/mL)	0.26	0.07
Limit of quantitation, LOQ (μg/mL)	0.80	0.20

### Limit of Quantitation (LOQ) and Limit of Detection (LOD)

The limit of quantitation (LOQ) was determined by establishing the lowest concentration that can be measured according to ICH Q2R1 recommendations [38] below which the calibration graph is non linear. The limit of detection (LOD) was determined by establishing the minimum level at which the analyte can be reliably detected [39].

$$\mathrm{LOQ}=10\;S_{a}/b\;\mathrm{LOD}=3.3\;S_{a}/b$$

Where S_a_ = standard deviation of the intercept of the calibration curve and b = slope of the calibration curve.

LOQ values were found to be 0.80, 0.20 μg/mL while LOD values were found to be 0.26, 0.07 μg/mL for LEV and AMB, respectively as shown in Table 2.

### Accuracy and precision

To prove the accuracy of the proposed method, the results of assay of the studied drugs were compared with those obtained using the comparison method [35]. Statistical analysis of the results obtained using Student's *t*-test and variance ratio F-test [38] revealed no significant difference between the performance of the two methods regarding the accuracy and precision, respectively Table 3.

**Table 3:** Assay results for the determination of LEV and AMB in pure form

**Compound**	**Proposed method**			**Comparison method (35)**
	**Amount taken (μg/mL)**	**Amount found (μg/mL)**	**% Found**	**% Found**
LEV	1.0	0.9897	98.97	99.35
	2.0	2.0161	100.81	100.96
	12.0	11.8548	98.79	99.49
	24.0	24.0249	100.10	
	28.0	28.1452	100.52	
	36.0	36.0484	100.13	
	44.0	43.8196	99.59	
$\bar{X}$ ± SD			99.84 ± 0.76	99.93 ± 0.89
*t*-test			0.16 (2.31)	
F-test			1.37 (5.14)	
AMB	1.0	1.0294	102.94	100.26
	4.0	3.9703	99.27	99.53
	12.0	11.9920	99.93	100.21
	16.0	16.0027	100.02	
	20.0	20.0134	100.07	
$\bar{X}$ ± SD			100.45 ± 1.43	100.00 ± 0.41
*t*-test			0.51 (2.45)	
F-test			12.31 (19.25)	

*N.B.* Each result is the average of three separate determinations.

The figures between parentheses are the tabulated t and F values at *P* = 0.05 [38].

The comparison method depends on using reversed phase HPLC for simultaneous determination of LEV and AMB using phosphate buffer – acetonitrile – methanol (650:250:100)v/v and pH adjusted to 5.2 with dilute orthophosphoric acid as the mobile phase and C18 column with UV detection at 220 nm [35]. The proposed procedure offers additional advantages over the comparison one in that the former is extended to the analysis of both drugs in human plasma. Moreover, using micellar mobile phase has the advantage of being low toxic due to the small amount of solvent employed. In addition, no need for pretreatment step for the analysis of human plasma.

Intra-day precision was assessed by analyzing three concentrations and three replicates of each concentration in one day. Also, the inter-day precision was assessed by analyzing three concentrations and three replicates of each concentration over three successive days. The relative standard deviations were found to be very small indicating reasonable repeatability and intermediate precision of the proposed method Table 4.

**Table 4:** Precision data for the determination of LEV and AMB by the proposed method

**Parameters**		**LEV concentration (μg/mL)**			**AMB concentration (μg/mL)**		
		**15.0**	**20.0**	**32.5**	**3.6**	**4.8**	**6.0**
**Intraday**	% Found	98.15	99.48	102.89	97.67	98.97	102.23
		97.00	98.82	100.93	102.99	102.06	101.01
		99.94	97.06	99.29	98.33	99.23	98.00
	$\left(\bar{X}\right)$	98.36	98.45	101.04	98.45	100.09	101.04
	± SD	1.48	1.25	1.80	1.25	1.71	1.80
	% RSD	1.51	1.27	1.78	1.27	1.71	1.78
	% Error	0.87	0.73	1.03	0.73	0.99	1.03
**Interday**	% Found	98.55	99.95	101.09	100.19	100.31	101.69
		98.92	99.03	99.20	99.22	102.03	100.51
		100.18	100.80	100.89	98.82	99.90	99.88
	$\left(\bar{X}\right)$	99.22	99.93	100.39	99.41	100.75	100.69
	± SD	0.86	0.89	1.04	0.70	1.13	0.92
	% RSD	0.86	0.89	1.04	0.71	1.12	0.91
	% Error	0.50	0.51	0.60	0.41	0.65	0.53

### Robustness of the method

The robustness of the proposed method was indicated by the constancy of the peak area ratio with deliberate changes in the experimental parameters. These parameters included n-propanol concentration, SDS concentration and pH of the mobile phase. These minor changes didn’t greatly affect the peak area ratios of both drugs.

### Selectivity

The selectivity of the method was investigated by observing any interference encountered from common tablet excipients. It was shown that these compounds did not interfere with the results of the proposed method. Additionally, there was not any interference encountered from human plasma matrix although no prior extraction procedure was performed.

### Applications

#### Analysis of LEV/AMB laboratory prepared mixtures

The proposed method was applied to the simultaneous determination of LEV and AMB in laboratory prepared mixtures in the recommended pharmaceutical ratios of 25:6 Figure 2. The concentrations of both drugs in the laboratory prepared mixtures were calculated according to the linear regression equations of the calibration graphs. The results obtained by the proposed method were in good agreement with those obtained using the comparison method [35]. The high percentage recoveries and the small values of the relative standard deviations and percentage relative errors indicate the high accuracy and precision of the proposed method, respectively. The results obtained are shown in Table 5. It was concluded that good recoveries were achieved for the studied drugs in their laboratory prepared mixtures.

**Table 5:** Assay results for the determination of LEV and AMB in their laboratory prepared mixture in 25:6 (w/w) as the case in tablets

**LEV/AMB ratio**	**Amount taken**		**Amount found**		**% Found**		**Comparison method (35)**	
	**(μg/mL)**		**(μg/mL)**					
	**LEV**	**AMB**	**LEV**	**AMB**	**LEV**	**AMB**	**LEV**	**AMB**
25:6	20.8	5.0	20.3840	5.0100	98.00	100.20	99.35	100.26
	25.0	6.0	24.4750	6.1680	99.00	102.80	100.96	99.53
	41.60	10.0	40.9050	10.1450	98.33	101.45	99.49	100.21
$\bar{X}$					98.44	101.48	99.93	100.00
± SD					0.61	1.30	0.89	0.41
% RSD					0.52	1.28	0.89	0.41
% Error					0.0.30	0.74	0.52	0.24
t					2.528			
F					3.064			

### Pharmaceutical application

#### Dosage form analysis

**Figure 3:**
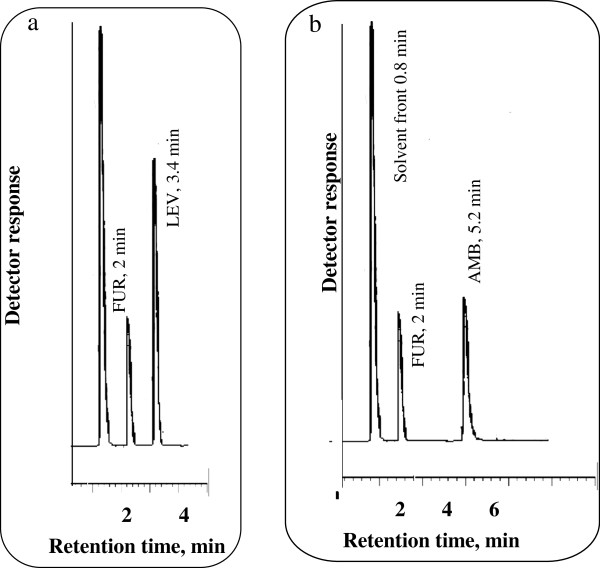
Chromatogram of (a) 25 μg/mL LEV in its single (Leeflox®) and (b) 6 μg/mL AMB in its single (Ambroxol®) tablet using 8 μg/mL FUR (I.S) in 0.15 M SDS, 0.3% triethylamine, 8% n-propanol all prepared in 0.02 M orthophosphoric acid at pH 4.0.

The proposed method was successfully applied to the assay of both LEV and AMB in their single tablets as illustrated in Figures 3a and b. The results of the proposed method were favorably compared with those obtained using the comparison method [35]. The results are abridged in Table 6. Statistical analysis of the results obtained using Student’s *t*-test and variance ratio F-test [38] revealed no significant difference between the performance of the two methods regarding the accuracy and precision, respectively Table 6.

**Table 6:** Assay results for the determination of LEV and AMB in their single tablet by the proposed and comparison methods

**Preparation**	**Proposed method**			**Comparison method (35)**
	**Amount taken**	**Amount found**	**% Found**	**% Found**
	**(μg/mL)**	**(μg/mL)**		
LeeFlox ^®^ tablets (250 mg LEV)	4.0	3.9180	97.97	100.10
	20.0	19.9980	99.99	99.02
	25.0	25.0100	100.04	98.16
$\bar{X}$ ± SD			99.31 ± 1.22	99.09 ± 0.97
% RSD			1.23	0.98
% Error			0.71	0.57
t-test			0.27 (2.78)	
F-test			1.47 (19.00)	
Ambroxol^®^ tablets (30 mg AMB)	4.0	4.0800	102.00	100.68
	8.0	8.2070	102.59	103.35
	16.0	16.0360	100.23	101.60
$\bar{x}$ ± SD			*101.60 ± 1.23	*101.88 ± 1.36
% RSD			1.21	1.33
% Error			0.70	0.77
t-test			0.26 (2.78)	
F-test			1.22 (19.00)	

*N.B.* Each result is the average of three separate determinations.

* Figures between parentheses are the tabulated t and F values, respectively at *p* = 0.05 [38].

Average LEV concentration found in Leeflox tablet 248.275 mg and average AMB found in Ambroxol tablet 30.48 mg.

**Figure 4:**
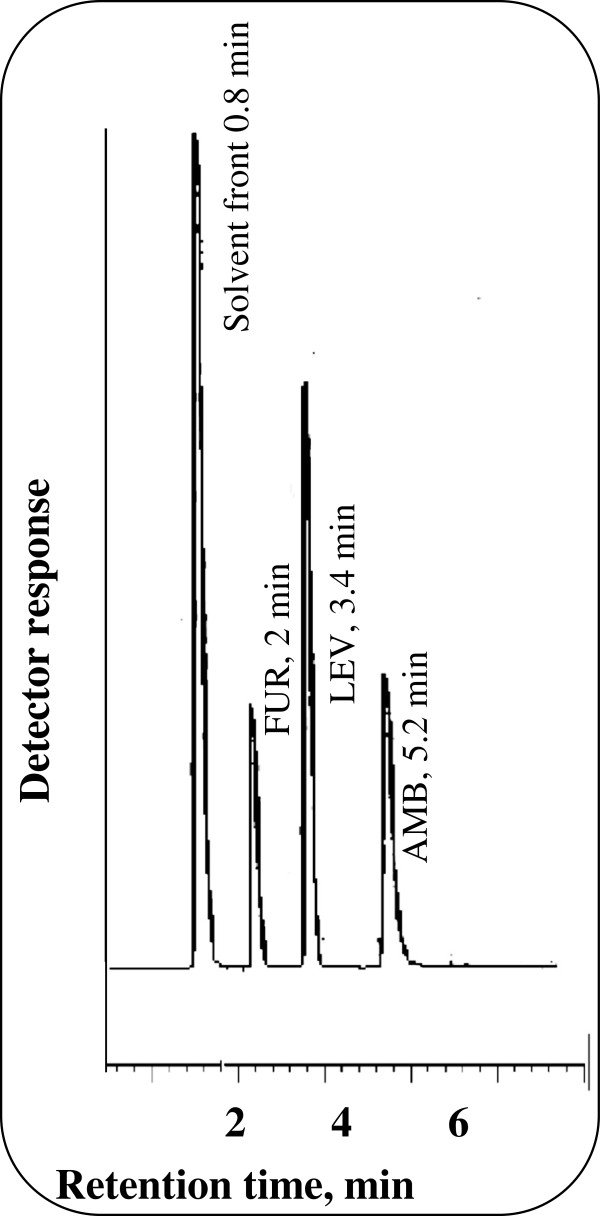
Typical chromatogram of co-formulated prepared tablet of 25 μg/mL LEV and 6 μg/mL AMB using 8 μg/mL FUR (I.S) in 0.15 M SDS, 0.3% triethylamine, 8% n-propanol all prepared in 0.02 M orthophosphoric acid at pH 4.0.

The proposed method was further applied to the determination of the studied drugs in their co-formulated laboratory prepared tablets. The results shown in Table 7 are in good agreement with those obtained with the comparison method [35]. Statistical analysis of the results obtained using Student’s *t*-test and variance ratio F-test [38] revealed no significant difference between the performance of the two methods regarding the accuracy and precision, respectively Table 8. Figure 4 shows chromatograms of good resolved peaks of LEV and AMB in their co-formulated tablets with high sensitivity.

**Table 7:** Assay results for the determination of LEV and AMB in their prepared tablets

**Preparation**	**Amount taken**		**Amount found**		**% Found**		**Comparison method (35)**	
	**(μg/mL)**		**(μg/mL)**					
	**LEV**	**AMB**	**LEV**	**AMB**	**LEV**	**AMB**	**LEV**	**AMB**
Prepared tablet (250 mg LEV+60 mg AMB)	5.0	6.0	25.1500	6.0020	100.60	100.04	100.10	100.68
	20.0	1.2	4.9200	1.2010	98.40	100.12	99.02	103.35
	25.0	4.8	19.7780	4.8250	98.89	102.00	98.16	101.60
$\bar{X}$					100.20*	100.72	99.09	101.88
**±** SD					±1.64	±1.11	±0.97	±1.36
% RSD					1.63	1.10	0.98	1.33
% Error					0.94	0.64	0.57	0.77
t					0.233	1.143		
F					1.418	1.495		

*N.B.* Each result is the average of three separate determinations.

The values of tabulated t and F tests are 2.78 and 19.00 respectively at *p* = 0.05 [38].

Average LEV concentration found in tablet 250.50 mg and average AMB found 60.14 mg per tablet.

**Table 8:** Assay results for the determination of LEV in spiked human plasma using the proposed method

**Parameter**	**Amount taken**	**Amount found**	**% Found**
	**(μg/mL)**	**(μg/mL)**	
LEV	1.0	1.0010	100.09
	2.0	2.0250	101.24
	3.0	2.9690	98.97
$\bar{x}$ ± SD	100.10 ± 1.14		
% RSD	1.13		
% Error	0.66		

### Application to biological fluid

After oral administration, LEV is rapidly absorbed with maximum plasma concentration being reached approximately one hour after a dose. It undergoes limited metabolism and is excreted mainly as the unchanged drug in urine (80-85%) and feaces (2%). About an oral administration of 100 mg dose of the drug, the mean plasma concentration was 1.35 mg/l and was observed after 1.8 hour after ingestion [40]. The high sensitivity of the proposed method allowed the determination of LEV in spiked human plasma.

### Analysis of spiked human plasma

**Figure 5:**
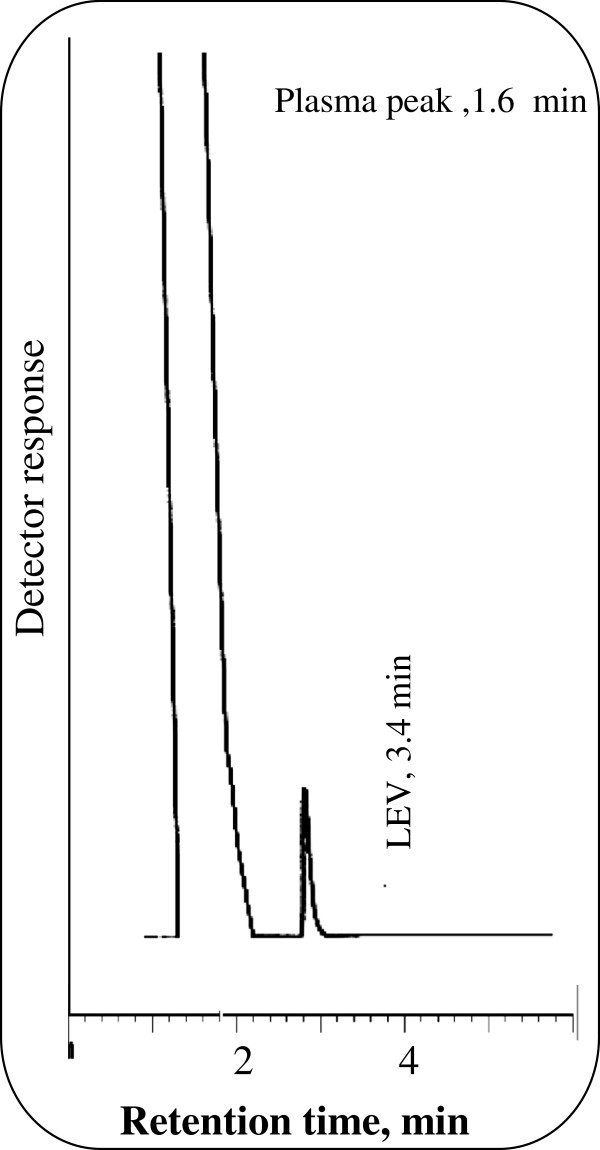
Application of the proposed method for the determination of LEV (2 μg/mL) in Spiked human plasma in 0.15 M SDS, 0.3% triethylamine, 8% n-propanol all prepared in 0.02 M orthophosphoric acid at pH 4.0.

The proposed method was applied for the determination of LEV in spiked human plasma without interfering from the plasma peak. Figure 5 shows LEV peak obtained from spiked human plasma. Table 8 shows the results obtained from spiked plasma. Under the above described experimental conditions, a linear relationship was established by plotting the peak area against the drug concentration in μg/mL due to overlapping between plasma peak and FUR (IS) peak. Linear regression analysis of the data gave the following equation:

$$P=\;‒\;139459+84957C\left(r=0.9999\right)$$

Where: P is the peak area, C is the concentration of the drug in μg/mL and r is the correlation coefficient.

The high value of the correlation coefficient (r) indicates the good linearity of the calibration graph constructed in human plasma.

### Real human plasma

**Figure 6:**
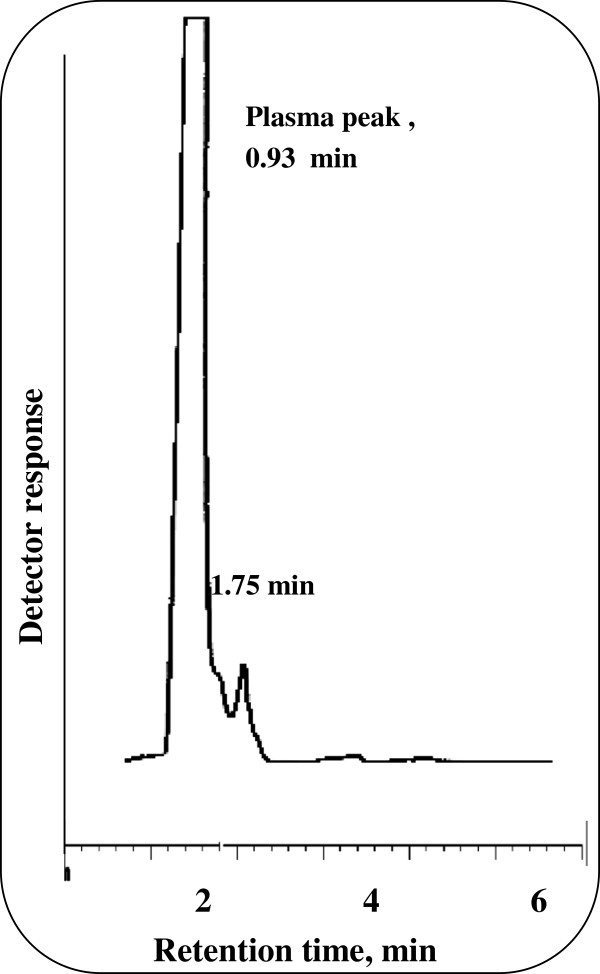
Blank plasma in 0.15 M SDS, 0.3% triethylamine, 8% n-propanol all prepared in 0.02 M orthophosphoric acid at pH 4.0.

**Figure 7:**
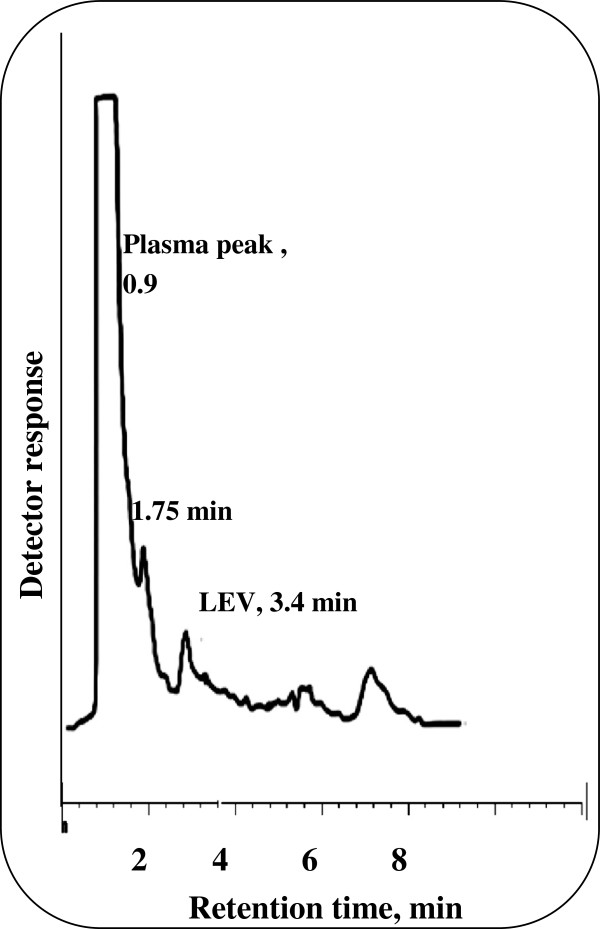
Application of the proposed method for the determination of LEV in real human plasma after 3 hours in 0.15 M SDS, 0.3% triethylamine, 8% n-propanol all prepared in 0.02 M orthophosphoric acid at pH 4.0.

**Figure 8:**
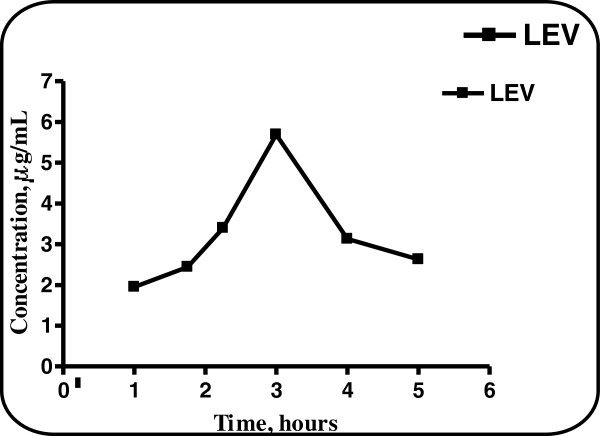
Monitoring of the blood level of in patient’s plasma at different time intervals.

The plasma samples obtained from the volunteer were investigated using the previously obtained calibration graph or regression equation of the spiked human plasma and the results obtained are shown in Figures 6 and 7. The mean plasma level reached after 3 hours for LEV and was found to be 5.69 μg/mL. Hence, the proposed method allows the therapeutic monitoring of the drug level in plasma Figure 8.

## Conclusion

A simple, accurate and rapid micellar less hazardous and toxic liquid chromatographic method was examined for the simultaneous determination of LEV and AMB in binary mixtures. The proposed method was successful in elution of LEV ad AMB with retention times at 3.4 min. and 5.2 min., respectively with a good resolution Rs = 3.81. The proposed method was found to have limits of detection of 0.26 and 0.07 μg/mL and limits of quantitation of 0.80 and 0.20 μg/mL for LEV and AMB, respectively that is more sensitive than the comparison method which is linear over the ranges of 7–22 μg/mL and 50–150 μg/mL for LEV and AMB, respectively. In addition, it could be applied to the analysis of both drugs in co-formulated tablets which wasn’t applicable in the comparison method. The good validation criteria of the proposed method allow its use in quality control laboratories. The proposed procedure, by virtue of its sensitivity, could be applied to the analysis of LEV in spiked human plasma with a mean recovery of 100.10 ± 1.14 without prior extraction procedure. In addition to the drug monitoring of LEV which gave a concentration of 5.69 μg/mL after 3 hours of oral administration of 750 mg of LEV. This seems to be promising in monitoring LEV level in patients undergoing LEV treatments for a long period.

## Abbreviations

LEV: Levofloxain hemihydrate; AMB: Ambroxol HCl; FUR: Furosimide; LOD: Limit of detection; LOQ: Limit of quantitation; BP: British pharmacopeia; USP: United States pharmacopeia.

## Competing interests

The authors declare that they have no competing interests.

## Authors’ contribution

FB suggested the proposed method and supervised the whole work, MK analyzed the data statistically, NM wrote the manuscript and reviewed the literature. SS carried out the experimental work, wrote down the data and carried out the calculations. All the authors read and approved the final manuscript.

## Authors' information

^1^ Professor and head of Analytical Chemistry Department, Faculty of Pharmacy, Mansoura University

^2^ Professor of Analytical Chemistry, Faculty of Pharmacy, Mansoura University

^3^ Professor of Analytical Chemistry, Dean of Faculty of Pharmacy, Mansoura University

^4^ Assistant lecturer, Analytical Chemistry Department, Faculty of Pharmacy, Mansoura University
